# Dialysis patients who smoke are more hypertensive, more fluid overloaded and take more antihypertensive medications than nonsmokers

**DOI:** 10.1080/0886022X.2020.1758723

**Published:** 2020-04-29

**Authors:** Mihály Tapolyai, Melinda Forró, Zsolt Lengvárszky, Tibor Fülöp

**Affiliations:** a Department of Dialysis, Hatvan Fresenius Medical Care Dialízis Központ, Hatvan, Hungary; b Ralph H. Johnson VA Medical Center, Charleston, SC, USA; c Department of Mathematics, Louisiana State University, Shreveport, LA, USA; d Department of Nephrology, Medical University of South Carolina, Charleston, SC, USA

**Keywords:** Bioimpedance, blood pressure, dialysis, fluid overload, overrhydration, smoking

## Abstract

**Background:**

Smoking remains a powerful risk factor for death in end-stage renal disease (ESRD) and so is the presence of fluid overload. The relationship between smoking, blood pressure (BP) control and volume overload is insufficiently explored in patients on maintenance dialysis.

**Methods:**

This is a retrospective cross-sectional cohort study, utilizing existing patients’ data generated during routine ESRD care, including bimonthly protocol bioimpedance fluid assessment of the volume status.

**Results:**

We analyzed the data of 63 prevalent patients receiving thrice weekly maintenance hemodiafiltration treatments at one rural dialysis unit in Hungary. The cohort’s mean ± SD age was 61.5 ± 15.3 years; 65% male, 38% diabetic, with a mean arterial blood pressure (MAP) 99.5 ± 16.8 mmHg and Charlson score 3.79 ± 2.04. Of these, 38 patients were nonsmokers and 25 smokers. The nonsmokers’ MAP was 94.3 ± 14.0 versus smokers’ 105.9 ± 18.9 mmHg (*p*: .002); nonsmokers took an average 0.73 ± 0.92 antihypertensive medications vs. 1.73 ± 1.21 for smokers (*p*: .0001). The distribution of taking more antihypertensive medications is skewed toward a higher number among the smokers (2x5 chi square *p*: .004). By bioimpedance spectroscopy, nonsmokers had an average 10.93 ± 7.65 percent overhydration (OH) over the extracellular space compared to 17.63 ± 8.98 in smokers (*p*: .005).

**Conclusions:**

Smoking may be a significant mediator of not only BP but also of chronic fluid overload in ESRD patents. Additional, larger studies are needed to explore the mechanistic link between smoking and volume overload.

## Introduction

The cardiovascular mortality rate of dialysis patients is about 10 to 20 times higher than that of the general population based on USRDS data [1]. The study by Foley reporting this excess risk [1] has been cited over 1600 times ever since 1998 to justify research and to investigate the cardiovascular component of illness in ESRD simply because the degree of increase in mortality is so astounding. Smoking represents a superimposed, additional layer of risk and been found to be a major cause of mortality in the dialysis population by others [2]. For patients with chronic kidney disease the risk of cardiovascular events was 36% higher among smokers than nonsmokers in a large (n: 6,245) observational study and the risk of death was increased by 48% (RR, 1.48; 95% CI, 1.30–1.70) [3]. Dialysis patients who smoked had an 65% higher hazard ratio for mortality in a meta-analysis [2] using the data of 26 studies. It appears, therefore, that smoking is a particularly toxic habit for those who have kidney disease.

Smoking seems to exert its deleterious effects through the acceleration of atherosclerotic disease. It is highly controversial whether hypertension as defined by high pre-HD blood pressure readings is an independent risk factor for mortality in HD patients – in fact, low pre-HD may be associated with higher mortality. The contribution of hypertension to the observed high risk in cardiovascular mortality in dialysis patients being uncertain, it is logical to look for additional mechanisms. In this study, we set out to investigate possible relationships between smoking and overhydration, an established independent predictor of increased cardiovascular mortality in this population. We devised a data review to see if smoking simply results in vasoconstriction or perhaps another mechanism may contribute to the development of hypertension and increased mortality.

## Methods

This is a cross sectional study of existing clinical data obtained through the clinical practice at a for-profit chain dialysis center in a rural environment in Hungary where the overall prevalence of smoking was 28% in 2014 [4]. The study was approved both by the commercial clinical provider (Fresenius Medical Care), as well as the medical Research Council of Hungary (equivalent to an independent review board), TUKEB permit number 27677-1/2019/EKU. Only aggregate data were utilized safeguards were in place to ensure patient anonymity and to prevent patient identification. All data collected were obtained as part of general practice and no additional data was used exclusively for research purposes.

We reviewed the data of 63 prevalent chronic patients receiving in-center on-line hemodiafiltration. All patients who had been dialyzed at our facility for at least 2 months were included in the study, with the only exception of those with missing demographic, bioimpedance or smoking status data. We used pre-dialysis BP’s for comparison of mean arterial BP’s of the cohorts and mid-week values for bioimpedance results. Mean arterial pressure (MAP) was calculated as MAP = 1/3 (SBP – DBP) + DBP.

We used *t* test to detect significance between the various parameters involving continuous data and we used Chi square test for categorical data. Multivariate regression analysis was also utilized when the associations determining fluid status and blood pressure (mean arterial pressure) were examined. Statistics were calculated using GraphPad 5.04 (San Diego, CA; USA) and figures were graphed using the same. Multivariate regression analysis was calculated by a professional mathematician to predict outcome of multiple categories using IBM SPSS (Armonk, NY; USA). We set the *p* value for significance to less than .05.

## Results

**Figure 1.**
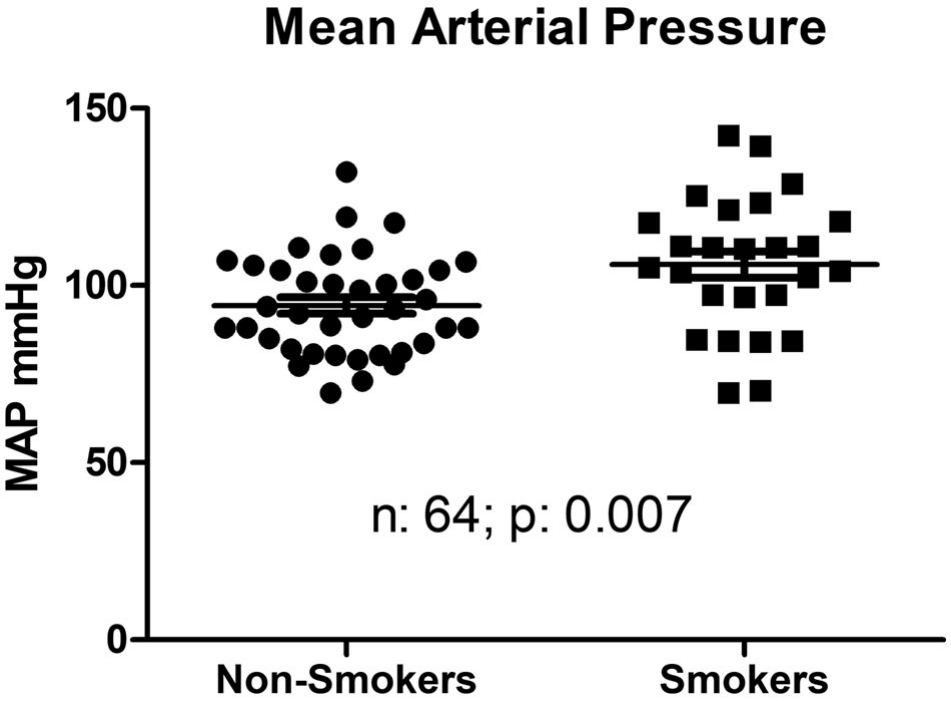
Pre-dialysis mean arterial blood pressure distribution between nonsmoker and smoker patients receiving thrice weekly hemodiafiltration.

**Figure 2.**
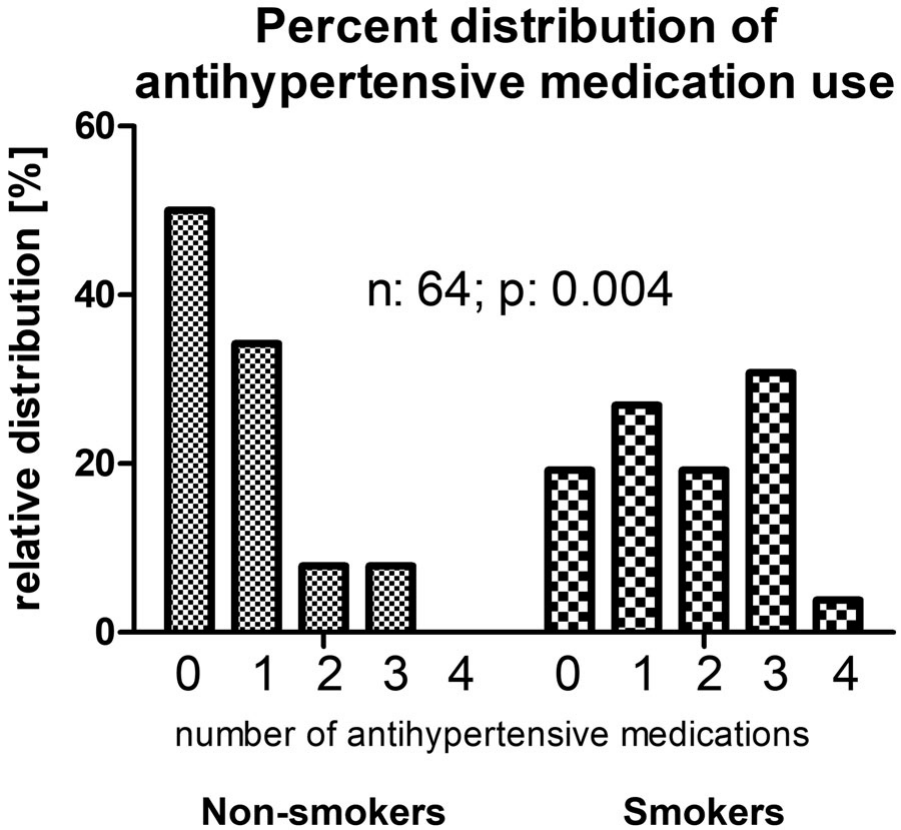
Histogram, depicting the percent distribution of the number of antihypertensive medications taken by nonsmoker and smoker patients receiving hemodialfiltration.

**Figure 3.**
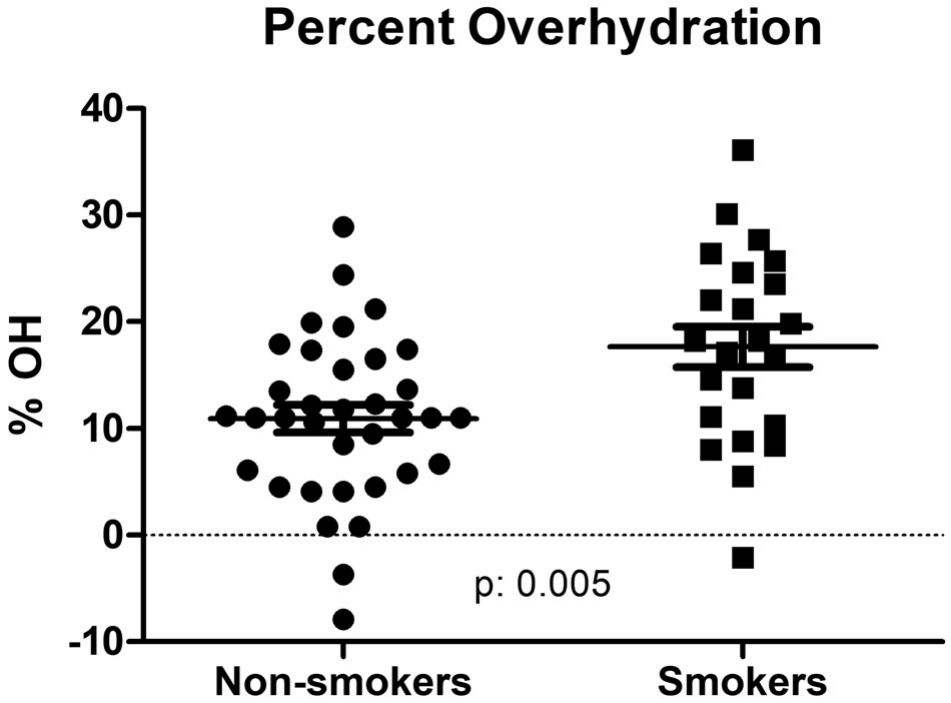
Percent overhydration among nonsmoker and smoker patients (n: 58) receiving hemodiafiltration.

Patients’ demographic data are displayed in Table 1. Briefly, the mean ± SD age of the smokers’ cohort was 61.5 ± 15.3 years, 65% male, 38% diabetic, MAP 99.5 ± 16.8 mmHg and Charlson score 3.79 ± 2.04. Of these, 38 patients were nonsmokers and 25 smokers. Blood pressure data and bioimpedance measurements are displayed in Table 2. The nonsmokers’ MAP was 94.3 ± 14.0 versus smokers’ 105.9 ± 18.9 mmHg (*p*: .002); systolic/diastolic pressures 146/68 ± 24/151 for nonsmokers 160/80 ± 15/73 mmHg for smokers (*p*: .02 for systolic and *p*: .001 for diastolic values). Figure 1 shows nonsmokers took an average 0.73 ± 0.92 antihypertensive medications vs. 1.73 ± 1.21 for smokers (*p*: .0001). The distribution of antihypertensive medications taken is skewed toward a higher number among the smokers (2 × 5 chi square *p*: .004). While 50% of nonsmokers took no antihypertensive medications at all, this rate was only 19% among the smokers. At the same time, only 7.9% (3 of the 38) nonsmokers took 3 antihypertensive medications and none took 4; the smokers’ proportion of taking 3 antihypertensive medication was 30.8% (8 of 25) and one patient was even on 4 antihypertensive medications in this group (Figure 2). Most importantly, nonsmokers had an average 10.93 ± 7.65 percent overhydration (OH%) by bioimpedance spectroscopy versus smokers’ relative overhydration was 17.63 ± 8.98 percent over the extracellular water space (*p*: .005) (Figure 3).

**Table 1.** Demographics.

	Entire cohort	Nonsmokers (*n*: 38)	Smokers (*n*: 25)	*p*
Male (%)	65	55	80	.044
Age (years)	61.5 ± 15.3	68.6 ± 13.9	50.7 ± 10.3	<.0001
Diabetic (%)	38.1	39.5	36.0	.785
Vintage (months)	77.1 ± 71.3	71.2 ± 60.5	85.9 ± 85.7	.428
Charlson score	3.79 ± 2.04	3.89 ± 2.12	3.64 ± 1.95	.633
Hemoglobin (g/dL)	10.9 ± 1.5	10.8 ± 1.5	11.2 ± 1.6	.248
OCM-Kt/V	1.68 ± 0.33	1.72 ± 0.32	1.61 ± 0.33	.175
Residual urine volume [mL]	460.9 ± 561.8	453.1 ± 562.3	471.7 ± 573.4	.904
NH Weight (kg)	81.3 ± 23.0	85.8 ± 24.9	74.3 ± 18.1	.060

Continuous variables as Means ± SD; OCM-Kt/V: On-line Clearance Measurement Kt/V; NH Weight: Normohydration Weight as determined by the bioimpedance.

**Table 2.** Results.

	Entire cohort	Nonsmokers (*n*: 38)	Smokers (*n*: 25)	*p*
Systolic BP (mmHg)	151.7 ± 24.2	146.3 ± 21.5	160.0 ± 26.0	.026
Diastolic BP (mmHg)	73.3 ± 15.9	68.3 ± 13.6	80.9 ± 16.4	.001
MAP (mmHg)	99.5 ± 16.8	94.3 ± 14.0	107.3 ± 17.8	.002
Number of medications	1.1 ± 1.1	0.7 ± 0.9	1.8 ± 1.1	<.0001
Overhydration (Liters)	2.86 ± 2.74	2.58 ± 3.22	3.28 ± 1.73	.344
Percent overhydration	13.6 ± 8.7	10.9 ± 7.6	17.6 ± 8.9	.003
TBW (L)	37.9 ± 8.6	37.6 ± 9.2	38.4 ± 7.9	.730
ECW (Liters)	19.1 ± 4.2	19.1 ± 4.6	19.0 ± 3.6	.921
ICW (Liters)	18.8 ± 4.8	18.4 ± 4.8	19.4 ± 4.8	.476
LTM (kg)	36.1 ± 10.2	34.1 ± 9.3	39.2 ± 10.9	.067
Relative LTM (%)	44.7 ± 11.8	40.4 ± 9.8	51.2 ± 11.7	<.0001
Fat (kg)	32.3 ± 13.8	36.6 ± 9.8	25.7 ± 10.3	.002
Relative fat (%)	37.7 ± 8.9	41.2 ± 7.6	32.4 ± 8.2	.0001

Actual values of variables (mean ± SD) examined in the entire cohort then the two separate cohort; *p* values calculated by Student’s t test. TBW: Total; Body Water; ECW: Extracellular Water; ICW: Intracellular Water; LTM: Lean Tissue Mass.

**Figure 4.**
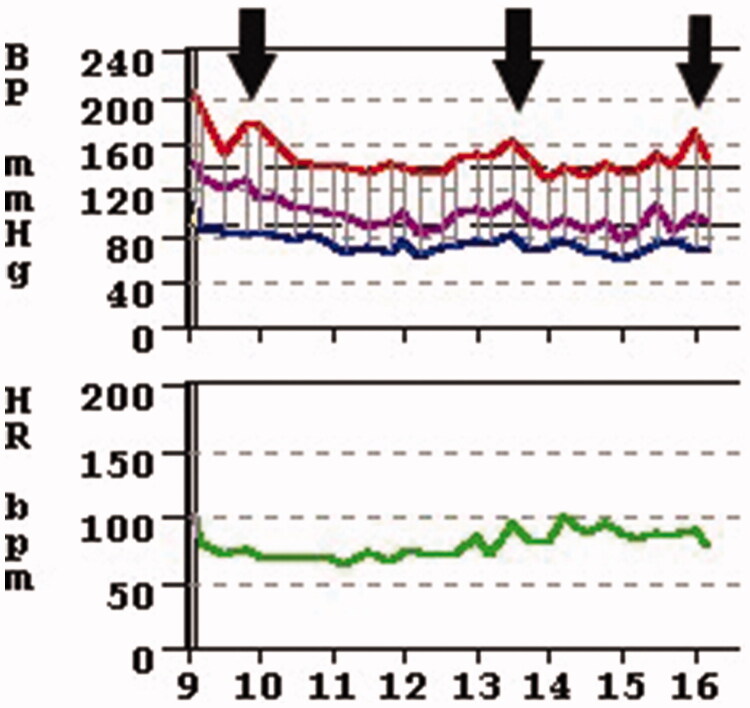
The author’s own observation on an 8-h ABPM (Ambulatory Blood Pressure Monitoring) revealing an acute blood pressure elevation at 09:30, 13:30 and 16:00, the exact time when the patient indicated that she smoked a cigarettes, Also note, that incidentally a severe white coat effect is also observable as the initial blood systolic pressure is over 200 mmHg yet the sustained blood pressure is around 150 mmHg.

Table 1 also shows that the smokers’ cohort was almost 18 years younger, 80% were male as opposed to 55% male in the nonsmoker group. Furthermore, Table 2 shows that their relative muscle mass (lean tissue mass) was greater and relative fat mass was smaller. We also performed a multivariate regression analysis for the determinants of percent-overhydration and blood pressure medications. On multivariate regression analysis, smoking emerged as the sole significant factor associated with increased antihypertensive medications use while no other factors including such as age, dialysis vintage, diabetic status, Charlson score of comorbidity, hemoglobin and online-measured (OCM) Kt/V reached statistical significance. The multivariate coefficient was 1.213 to a significant degree (*p*: .001) for smoking. As shown in Table 3, smoking status was the only significant factor predicting overhydration. The multivariate regression coefficient for percent overhydration (OH%) (Table 3) as a dependent variable was 9.905 and it was statistically significant (*p*: .001) while all other above-mentioned independent variables’ multivariate coefficients were not statistically significant. For the MAP only age had a statistically significant (<.0001) bearing with a coefficient of −0.605 but not smoking. For absolute lean tissue mass (LTM) smoking had not an influence and for the relative LTM the multivariate coefficient was not significant at 6.076 (*p*: .087). For the relative LTM% the only significant (*p*: .035) coefficient was for the diabetic status −7.747. For absolute fat mass the multivariate regression coefficient was of −13.321 (*p*: .005) and this significance remained present for percent fat mass at −6.506 (*p*: .02). Though these issues regarding fat and lean tissue mass were not the subject of our investigation, there may be a well-recognized association between smoking and fat mass. Figure 4 shows the ambulatory blood pressure monitoring (ABPM) tracing of a patient who smoked while wearing the monitor thus giving us an accidental opportunity to observe what actually happens to blood pressure while smoking compared to the rest of the time on ABPM.

**Table 3.** Multivariate regression analysis results of a model for pre-dialysis percent overhydration using predictors in the first column.

	Unstandardized Coefficients		Standardized Coefficients		
Model	B	Std. Error	beta	*t*	Significance
(constant)	1.36	12.659		0.107	.915
Male gender	−1.73	2.391	−0.094	−0.723	.473
Age (years)	0.07	0.089	0.113	0.727	.471
DM	4.37	2.832	0.246	1.543	.129
Smoking status	9.91	2.765	0.557	3.582	.001
Vintage (months)	−0.01	0.016	−0.044	−0.339	.736
Charlson score	0.19	0.626	0.044	0.297	.767
Hemoglobin	−0.88	0.734	−0.163	−1.192	.239
OCM-Kt/V	7.67	3.953	0.281	1.932	.059

OCM-Kt/V: on-line Kt/V measurement results.

## Discussion

Smoking causes blood pressure elevation by several mechanisms including an impairment of endothelium-dependent vasodilation [5] and endothelial injury [6]. Smoking also causes acute increases in norepinephrine and epinephrine causing acute blood pressure elevation [7] (Figure 4) and an increase of sympathetic outflow [8]. Smoking also alters the vascular shear force and blood rheology thus exacerbating vascular damage [9,10] and a risk factor for chronic kidney disease in animal models [11]. Nicotine containing chewing gums, transdermal patches and IV infusion of nicotine all cause acute blood pressure elevations [10] thus nicotine can be implicated in the smoker dialysis patients’ pre-dialysis blood pressures which we found to be significantly higher than others. While nicotine may be one of the important toxins of cigarette smoking carbon monoxide may be another more important toxin [12], having the greatest concentration at the time of smoking. These studies cited above and also our ABPM tracing of a non-dialysis patient in Figure 4 indicate that this effect of acute blood pressure elevation might be generalizable to both the dialysis and non-dialysis population. The blood pressures tallied for this study were all pre-dialysis BP’s. Furthermore, all patients on dialysis who smoke were interviewed and asked whether they did smoke a cigarette just before entering the dialysis suite’s waiting room and 100% of them gave an affirmative answer. They admitted that they always smoke before their dialysis sessions; again, indicating the possibility that a single cigarette just before dialysis may be sufficient to raise pre-dialysis blood pressure. This may actually partially explain their higher BP as well as why they are on more antihypertensive medications and ultimately more fluid overloaded. Smoking has been a well-established risk factor for congestive heart failure, one manifestation of fluid overload in the non-dialysis, general population [13] as well as in the dialysis population at dialysis initiation due to refractory congestive heart failure [14]. We believe there might be a different mechanism at play in this special, dialysis cohort. Our working theory is that the rounding physicians are more likely to prescribe antihypertensive medications to these more severely hypertensive patients at the beginning of their dialysis sessions. However, we have shown that escalating antihypertensive medications in the dialysis population does not ameliorate blood pressure control [15] this approach may actually contribute to the preservation of hypertensive status. There may be many reasons why this happens but we speculated that antihypertensive medications prevent adequate fluid removal during. We found by bioimpedance measurements that the polypharmatized dialysis patients’ number of antihypertensive medications – including diuretics – had a strong (*r*: 0.54 *p*: <.0001) correlation with their fluid overload [16]. Patients with multiple antihypertensive medications run the risk of not tolerating adequate fluid removal even though “avoiding medication-directed control of BP may enhance the opportunity to probe dry-weight, facilitate removal of volume, and limit the risk for pressure-volume overload, which may be a significant concern” [17]. Thus, it seems that pre-dialysis smoking initiates a spiral of acute blood pressure elevation that prompts physicians to prescribe antihypertensive medications that prevent adequate fluid removal during dialysis because of intradialytic hypotension and pharmacological blockade of the physiological response to fluid loss. This may further exacerbate smoker dialysis patients’ hypertension because they will be more fluid overloaded. Others [18] have also found that dialysis patients who smoke have more lung water when measured by lung ultrasound comets, perhaps due to the same mechanism. We examined the possibility that the phenomenon of fluid excess among smokers may simply be the result of the smokers’ demographics, namely that smaller patients, young people and males have been shown [16] to have more fluid overload, however, the multivariate regression model failed to confirm this. Notwithstanding, our finding of fluid overload among smokers is somewhat of a surprise and may need further exploration as to why or by what mechanism do smokers become fluid overloaded more than nonsmokers. We acknowledge that there may be a number of other mechanisms affecting the pre-dialysis blood pressure such as increased thirst, anxiety, apprehension etc., we put forward speculation of a mechanism as above.

While it is admittedly the weakness of the study that the total number of dialysis patients is relatively low, the statistical strength of our findings with *p* values less well below .01 makes us confident of the validity of the study. This is an observational, retrospective study, not a trial; therefore a cause and effect association could not be stated. We still feel, however, that these observations, namely that smoking may be associated with fluid overload are clinically important findings. We conclude that smoker dialysis patients’ blood pressure may be more volume-related than that of nonsmokers in addition to the other deleterious effects of smoking.

## Data Availability

De-identified data used to support the findings of this study are available from the corresponding author upon request.

## References

[1] Foley RN, Parfrey PS, Sarnak MJ. Epidemiology of cardiovascular disease in chronic renal disease. J Am Soc Nephrol. 1998;9(12 Suppl):S16–S23. 11443763

[2] Liebman SE, Lamontagne SP, Huang LS, et al. Smoking in dialysis patients: a systematic review and meta-analysis of mortality and cardiovascular morbidity. Am J Kidney Dis. 2011;58(2):257–265. 21664017 10.1053/j.ajkd.2011.03.025 PMC3247014

[3] Staplin N, Haynes R, Herrington WG, et al. Smoking and adverse outcomes in patients with CKD: The Study of Heart and Renal Protection (SHARP). Am J Kidney Dis. 2016;68(3):371–380. 27118687 10.1053/j.ajkd.2016.02.052 PMC4996629

[4] Cselkó Z, Kovács G, Horváth I. The smoking situation in Hungary. Tob Induc Dis. 2018;16(1):100–100.

[5] Yugar-Toledo JC, Ferreira-Melo SE, Sabha M, et al. Blood pressure circadian rhythm and endothelial function in heavy smokers: acute effects of transdermal nicotine. J Clin Hypertens (Greenwich). 2005;7(12):721–728. 16330894 10.1111/j.1524-6175.2005.04597.x PMC8109293

[6] Pittilo RM, Bull HA, Gulati S, et al. Nicotine and cigarette smoking: effects on the ultrastructure of aortic endothelium. Int J Exp Pathol. 1990;71(4):573–586. 2400739 PMC2002282

[7] Cryer PE, Haymond MW, Santiago JV, et al. Norepinephrine and epinephrine release and adrenergic mediation of smoking-associated hemodynamic and metabolic events. N Engl J Med. 1976;295(11):573–577. 950972 10.1056/NEJM197609092951101

[8] Narkiewicz K, van de Borne PJ, Hausberg M, et al. Cigarette smoking increases sympathetic outflow in humans. Circulation. 1998;98(6):528–534. 9714109 10.1161/01.cir.98.6.528

[9] Powell JT. Vascular damage from smoking: disease mechanisms at the arterial wall. Vasc Med. 1998;3(1):21–28. 9666528 10.1177/1358836X9800300105

[10] Benowitz NL, Gourlay SG. Cardiovascular toxicity of nicotine: implications for nicotine replacement therapy. J Am Coll Cardiol. 1997;29(7):1422–1431. 9180099 10.1016/s0735-1097(97)00079-x

[11] Arany I, Taylor M, Fulop T, et al. Adverse effects of chronic nicotine exposure on the kidney: Potential human health implications of experimental findings. CP. 2018;56(11):501–506. 10.5414/CP203302 30148451

[12] Benowitz NL. The role of nicotine in smoking-related cardiovascular disease. Prev Med. 1997;26(4):412–417. 9245658 10.1006/pmed.1997.0175

[13] Suskin N, Sheth T, Negassa A, et al. Relationship of current and past smoking to mortality and morbidity in patients with left ventricular dysfunction. J Am Coll Cardiol. 2001;37(6):1677–1682. 11345383 10.1016/s0735-1097(01)01195-0

[14] Fabbian F, Cantelli S, Molino C, et al. Dialysis initiation and survival in patients with refractory congestive heart failure. Int J Artif Organs. 2009;32(8):492–495. 19844893 10.1177/039139880903200803

[15] Tapolyai M, Karim J, Fakhruddin A. Escalating antihypertensive medications in end‐stage renal disease patients does not improve blood pressure control. J Clin Hypertension. 2008;10(3):215–218. 10.1111/j.1751-7176.2008.07198.x PMC8110059 18326963

[16] Tapolyai M, Faludi M, Reti V, et al. Dialysis patients’ fluid overload, antihypertensive medications, and obesity. Asaio J. 2011;57(6):511–515. 21989422 10.1097/MAT.0b013e3182377216

[17] Agarwal R, Weir MR. Dry-weight: a concept revisited in an effort to avoid medication-directed approaches for blood pressure control in hemodialysis patients. CJASN. 2010;5(7):1255–1260. 20507951 10.2215/CJN.01760210 PMC2893058

[18] Mallamaci F, Benedetto FA, Tripepi R, et al. Detection of pulmonary congestion by chest ultrasound in dialysis patients. JACC Cardiovasc Imaging. 2010;3(6):586–594. 20541714 10.1016/j.jcmg.2010.02.005

